# Improving patient self-reporting of antihypertensive adverse drug events in primary care: a stepped wedge cluster randomised trial

**DOI:** 10.1186/s12875-021-01478-w

**Published:** 2021-08-07

**Authors:** Karine Buchet-Poyau, Pauline Occelli, Sandrine Touzet, Carole Langlois-Jacques, Sophie Figon, Jean-Pierre Dubois, Antoine Duclos, Marc Chanelière, Cyrille Colin, Muriel Rabilloud, Maud Keriel-Gascou

**Affiliations:** 1 Hospices Civils de Lyon, Pôle de santé publique, Service Recherche et Epidémiologie Clinique, F-69003 Lyon, France; 2 Laboratoire Research on Healthcare Performance RESHAPE, INSERM U1290, Université Lyon 1, F-69622 Villeurbanne, France; 3 Hospices Civils de Lyon, Pôle de santé publique, Service de Biostatistique, F-69002 Lyon, France; 4 Collège universitaire de Médecine Générale, Université Lyon 1, F-69622 Villeurbanne, France; 5 Hospices Civils de Lyon, Pôle de santé publique, Service des Données de Santé, F-69003 Lyon, France; 6 Hospices Civils de Lyon, Pôle de santé publique, Service d’Evaluation Economique en Santé, F-69003 Lyon, France; 7 CNRS, UMR 5558, Laboratoire de Biométrie et Biologie Evolutive, Equipe Biostatistique Santé, Université Lyon 1, F-69622 Villeurbanne, France

**Keywords:** Patient safety, Patient-centred care, Family medicine, Adverse drug events, Antihypertensive drugs, Educational booklet, Stepped wedge cluster trial

## Abstract

**Background:**

About 25% of patients experience adverse drug events (ADE) in primary care, but few events are reported by the patients themselves. One solution to improve the detection and management of ADEs in primary care is for patients to report them to their general practitioner. The study aimed to assess the effect of a booklet designed to improve communication and interaction between patients treated with anti-hypertensive drugs and general practitioners on the reporting of ADEs.

**Methods:**

A cluster randomized controlled cross-sectional stepped wedge open trial (five periods of 3 months) was conducted. A cluster was a group of general practitioners working in ambulatory offices in France. Adults consulting their general practitioner to initiate, modify, or renew an antihypertensive prescription were included. A booklet including information on cardiovascular risks, antihypertensive treatments, and ADE report forms was delivered by the general practitioner to the patient in the intervention group. The primary outcome was the reporting of at least one ADE by the patient to his general practitioner during the three-month period after enrolment. Two clusters were randomised by sequence for a total of 8 to receive the intervention. An intention-to-treat analysis was conducted. A logistic mixed model with random intercept was used.

**Results:**

Sixty general practitioners included 1095 patients (median: 14 per general practitioner; range: 1–103). More patients reported at least one ADE to their general practitioner in the intervention condition compared to the control condition (aOR = 3.5, IC^95^ [1.2–10.1], *p* = 0.02). The modification and initiation of an antihypertensive treatment were also significantly associated with the reporting of ADEs (aOR = 4.4, CI^95^ [1.9–10.0], *p* <  0.001 and aOR = 11.0, CI^95^ [4.6–26.4], *p* <  0.001, respectively). The booklet delivery also improved patient satisfaction on general practitioner communication and high blood pressure management.

**Conclusion:**

A booklet can improve patient self-reporting of ADEs to their general practitioners. Future research should assess whether it can improve general practitioner management of ADEs and patient’s health status.

**Trial registration:**

Trial registry identifier NCT01610817 (2012/05/30).

## Background

About 25% of patients experience adverse drug events (ADEs) in primary care [1–3] and over a quarter of these could be prevent if situations at risk were detected earlier [4]. Event severity could also be reduced if patients were better engaged in self-reporting to their general practitioner (GP) [4].

Antihypertensive drugs are amongst the main classes of drugs responsible for ADEs in primary care. Between 11 and 14% of antihypertensive treated patients experience at least an ADE [1, 2, 4]. Of note, different classes of antihypertensive drugs are often prescribed together or in combination with other drugs, increasing the risk of ADEs [5, 6].

It has been shown that ADEs reported by patients are complementary to those reported by professionals [7, 8]. However, patients report ADEs four times less frequently than healthcare professionals [8]. Collecting ADEs from a patient’s perspective is critical in reducing their occurrence, which could result in warranted and unwarranted healthcare utilisation, and ultimately rendering care more patient centred [7]. Hence, it seems important to encourage patients to report ADEs to their GP, particularly patients treated with antihypertensive drugs who are at high risk of ADEs.

It has been shown that information booklets can improve illness and drug management, reduce the number of medical encounters, and improve patient knowledge and drug adherence [9–15]. In these previous studies however, patients were not encouraged to actively contribute to ADE reporting. A patient-centred booklet was therefore developed to improve communication and interaction between the patient and his GP on antihypertensive drug management [16]. The present study aimed at assessing the impact of this booklet on patient self-reporting of ADEs to their GPs.

## Methods

### Trial design

The study was a cross-sectional stepped wedge cluster randomized trial [17, 18].

Eighty-eight GPs accepted to participate and were grouped into eight clusters based on geographic proximity. Five periods of 3 months were defined. During the first period, all clusters belonged to the control condition. After that, the intervention was rolled out sequentially two clusters at a time, and the order in which the clusters joined the intervention condition was randomized [17]. No major changes were made after the start of the trial.

### Setting and participants

The patients were included (for 3 months) by GPs successively between June 2012 and September 2013, in the *Auvergne-Rhône-Alpes* and *Ile-de-France* regions in France. Inclusion criteria were: patients over 18 years old who were consulting to initiate, modify, or renew an antihypertensive prescription. Patients had to agree to answer a telephone survey at home and to understand the French language [17].

### Intervention condition

The intervention consisted in a booklet designed as a communication tool between the patient and his GP [16, 17]. The aim of this booklet, designed as a support of exchange between patient and GPs, is to improve patient self-reporting of ADE to GPs, patient knowledge on antihypertensive drug management and patient satisfaction on GP communication about their care. It was developed following a recommended 10 step-process (Table 1 [16];). It included information on cardiovascular risks and management of antihypertensive treatments, care plans, and ADE report forms that could be annotated by the patient (Table 2). The way to deliver the booklet to the patient was explained to the GPs during a webseminar. They had to evaluate patient knowledge concerning their antihypertensive prescription, explain the booklet to the patient, write the prescriptions and lab tests with the patient, and finally explain how to use the ADE report forms [17].

**Table 1:** The 10 step-process of booklet development (see reference [16])

N°	Steps	Participants
**1**	**Literature review** - Selection of items on high blood pressure management and the use of antihypertensive drugs	**Work group A**: General practitioners (4), public health practitioners (2)
**2**	**First round of Delphi survey (27 experts) -** First validation of selected items for the booklet	**Work group B**: Patients (2), general practitioners (11), public health practitioners (2), cardiologist (1), geriatrician (2), psychologist (1), economist (1), pharmacists (2), nurse (1) ethicist (1), pharmacovigilance practitioners (2) and internist (1)
**3**	Development of the initial layout of the booklet	**Work group A**
**4**	**Second round of Delphi survey** - Reiteration of equivocal items	**Work group B**
**5**	**Third round of Delphi survey** - Achieving expert consensus on items *Validation of the 1rst version of the booklet*	**Work group B**
**6**	**Independent review of the booklet** - Certification of the conformity of the given information with current scientific data and simplification of medical terminology	Anthropologist (1) and independant general practitioner (1)
**7**	**Readability assessment** (Rudolf Flesch test) and Computer graphics work *Validation of the 2nd version of the booklet*	**Work group A** and Computer graphics designer (1)
**8**	**Qualitative study in primary care** (Observation of consultations and semi-directed individual interviews of patients and general practitioners) **–** Assessment of understanding and acceptability of the booklet	General practitioners of various populations of patients (7), healthcare assistants (2) and patients (13). Patients from 44 to 86 y: employees (6), liberal profession (1), labourers (4), craftsman (1), intermediate profession (1)
**9**	**Linguistic work** - in accordance with the principles of controlled language and understandable by the target population	Linguist (1) and **Work group A**
**10**	Computer graphics work ***Validation of the 3rd version of the booklet***	Computer graphics designer (1) and **Work group A**

**Table 2:** Extract of the booklet designed to facilitate communication between patients and General Practitioners on the reporting of Adverse Drug Events (ADEs)

**Title page**	
Hypertension: control your blood pressure, prevent ADEs. A guide linking patient and caregiver	
**Instructions for use of the booklet**	
1) Your doctor informs you (...). Together you write your care plan. 2) At each consultation: bring your guide (...). 3) In case of an adverse event: fill in the report form (...)	
**Control your blood pressure**	
- Hypertension and cardiovascular risk - How can you reduce your cardiovascular risk? - What are the benefits of antihypertensive drugs?	
**Prevent ADEs**	
- Be careful in certain situations - Beware of drug combinations - What are the possible side effects of antihypertensive medications?	
**Your care plan (to be written with the help of your doctor)**	
- Your objectives for the next visit regarding your habits (tobacco, alcohol, physical activity, salt, sugars, fats) - Your treatments - Stopped or contraindicated antihypertensive drugs - Examinations to be done prior to the next visit - Points to discuss with your doctor	
**Reporting an ADE**	
Fill in this form and contact your doctor as soon as possible - manifestations of ADE (date, time, signs) - suspected drug (antihypertensive or not) - Following the adverse reaction, what did you do to mitigate the ADE?	

### Control condition

Patients included in the control condition did not receive the booklet. Care management was carried out according to the GP’s usual practice for antihypertensive-treated patients, but remained heterogeneous [19].

### Sample size

Sample size was determined using the Hussey and Hughes method [20] to detect a 4.5% increase in the percentage of patients who actively self-report at least one ADE to his GP i.e. an increase from 3% in the control condition to 7.5% in the intervention condition. The inclusion of 30 patients per cluster and period (a total of 1200 patients) would allow to reach a power of at least 80% for inter-cluster coefficient of variation of 0.5 and a bilateral type I error of 5% (see protocol [17] for the sensitivity analysis).

### Randomization

The order in which the clusters of GPs integrated the intervention condition was randomized using the procedure PROC PLAN of software SAS, version 9.2 (Copyright (c) 2002–2012 by SAS Institute Inc., Cary, NC, USA). This was performed by a biostatistician, independent from the coordinating centre. GPs were informed of their date of allocation to the intervention condition 1 month prior to the date of intervention implementation to organise their training.

### Primary and secondary outcomes

All outcome measures pertain to the patient level.

The primary outcome was the reporting of at least one ADE by the patient to his GP during the three-month period after enrolment (patient self-reported ADE).

The secondary outcome was the reporting of at least one ADE by the patient and/or his GP.

An ADE was defined as a deleterious effect associated with a drug, reported by the patient and/or the GP, and validated by a study committee. ADEs could involve all drugs taken by the patients. The study committee composed of a pharmacologist, a GP (KGM), and the project manager (BPK), validated each ADE, classified its seriousness, the associated symptoms, associated drugs.

Other secondary outcomes were patient knowledge about cardiovascular risk factors and risk associated with hypertensive drug, patient satisfaction concerning the GP’s ability to communicate, and the information provided by the GP on high blood pressure.

### Data collection

Morbidity, drugs, and situations at risk of antihypertensive drug-related ADEs were collected in an online form filled out by the GP at patient inclusion. At the end of the 3 months of each patient inclusion, the GP completed data on ADEs, their associated symptoms and drugs. Moreover, a research assistant, supervised by a general practitioner (MKG), called each patient to explore the onset of ADEs not reported to the GP. Patient socio-demographic data, patient knowledge about cardiovascular risk factors and prescribed antihypertensive drugs, as well as patient satisfaction concerning communication with his GP were also collected during the call.

### Blinding

Patients and GPs were not blinded to the assignment to sequences. The clinical research assistants, the statisticians, and the ADE study committee were blinded to the intervention for data collection, randomization and analysis, and validation of ADEs.

### Statistical analysis

Description concerned GP and patient characteristics, ADEs self-reported by patients to their GP, and all ADEs reported by patients and/or their GPs. The committee classified each ADE according to who reported it: ADEs reported by GPs in the study online form but not reported by patients to the research assistant, ADEs reported by patients to the research assistant but not by the GPs in the online form and ADEs reported both by the GPs in the online form and by their patients to the research assistant.

Patient characteristics were compared between the control and the intervention condition using Student t-test for quantitative characteristics and chi-squared test or Fisher’s exact test for qualitative characteristics.

The effect of the intervention on the primary outcome was analysed on an intention-to-treat basis. A logistic mixed model with random intercept was used to quantify the intervention effect on the probability that a patient self-reported at least one ADE to his GP. The model was adjusted on the time period introduced as an ordinal covariate in order to take into account a time trend. It was also adjusted on age, gender, educational level, morbidities, antihypertensive prescription (renewal, modification, or initiation) and situations presenting a risk of ADEs associated to antihypertensive drugs. The random effect on intercept was used to take into account a GP effect rather than a cluster effect. Indeed a first analysis showed the absence of outcome variability between clusters, while a significant variability existed between GPs. The effect of the intervention was quantified by an adjusted odds ratio (aOR) with its 95% confidence interval (CI). A similar analysis was carried out on the probability that at least one ADE was reported by a patient or his GP.

Analyses were carried out using the software SAS version 9.2.

We followed the CONSORT criteria from EQUATOR network to report the study. We have also taken into account the recommendations for stepped wedge studies [21].

## Results

### Characteristics of GPs

Out of the 88 GPs, 60 included patients and participated in the study throughout its duration (Table 3). Six dropped out the study before the beginning of the inclusion’s period and 22 did not include a patient. Their mean age was 51.7 (SD 9.2) years and they were 70.0% male. Among them, 55.0% worked in urban areas, 31.7% in peri-urban areas, and 13.3% in rural areas. A majority (76.6%) supervised students as tutors for their University.

**Table 3:**
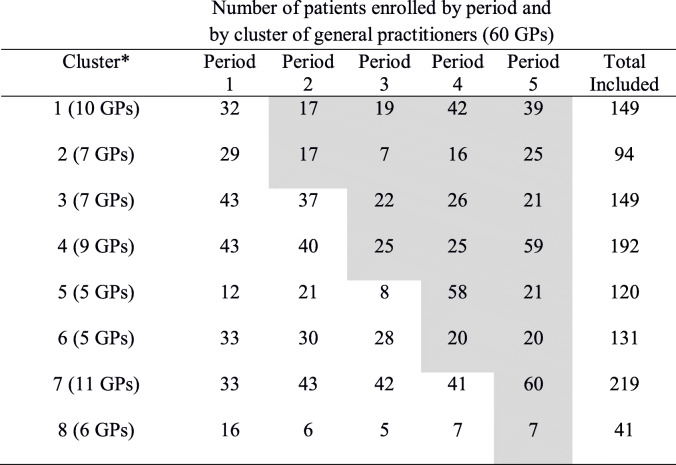
Presentation of the stepped wedge design and cluster sizes (*N* = 1095)

Grey cells are intervention condition periods

^a^Cluster: GPs were grouped according to geographic proximity or practice in the same medical centre

### Characteristics of patients

Among the 1102 patients included, 1095 patients were analysed, representing a median of 14 patients included per GP (range: 1–103). Reasons for exclusion were patients included twice and missing data on the main outcome. Patients from the intervention condition were significantly younger, more educated, with more situations presenting a risk of ADE but were less frequently exposed to at least two antihypertensive treatment classes. The antihypertensive prescriptions were not significantly different between both conditions (Table 4).

**Table 4:** Characteristics of patients at inclusion (*N* = 1095)

	Control (***N*** = 549)	Intervention (***N*** = 546)	***p-value***
Age, mean (SD)	64.7 (11.8)	63.2 (11.3)	**0.03**
Male, % (n)	51.7 (284)	54.6 (298)	0.35
Educational level^a^, % (n)			**0.03**
Secondary school or no diploma	40.7 (208)	33.4 (164)	
Vocational/High-school degree	38.8 (198)	40.3 (198)	
University degree	20.6 (105)	26.3 (129)	
Socio-professional category^a^, % (n)			0.09
Farmers, artisans, shopkeepers, CEOs	11.0 (56)	11.8 (58)	
Executives, intellectuals, and intermediate professions	30.3 (155)	35.6 (175)	
Employees and workers	53.2 (272)	49.5 (243)	
Unemployed, retired, students	5.5 (28)	3.1 (15)	
Morbidity ≥3 chronic pathologies ^a^, % (n)	8.6 (44)	6.8 (34)	0.28
Situations with a risk of ADEs ^a^, % (n)	8.5 (46)	15.4 (82)	**< 0.001**
Medications ≥4^a^, % (n)	54.8 (301)	49.6 (271)	0.09
mean (± SD)	4.3 (2.8)	4.2 (3.0)	
Antihypertensive classes ≥2^a^, % (n)	63.8 (350)	55.5 (303)	**0.01**
mean (± SD)	2.0 (0.9)	1.8 (0.9)	
Antihypertensive prescription^a^, % (n)			0.16
Renewal	89.2 (487)	85.4 (462)	
Modification	7.0 (38)	9.8 (53)	
Initiation	3.9 (21)	4.8 (26)	

*SD* standard deviation, *ADE* adverse drug event

Chronic pathologies: heart failure, heart rhythm disorder, anchor, myocardial infarction, obliterating arteriopathy of the lower limbs, renal artery stenosis, diabetes, cerebrovascular accident, COPD or chronic respiratory failure, asthma, cirrhosis or liver failure, renal failure (creatinine clearance < 60 ml/min), gout, cancer, depression, Parkinson

Situations presenting a risk of antihypertensive drug-related ADEs: hypotension, fever, nutritional depletion, diarrhoea, nausea, vomiting, abdominal pain, heat wave, reduced mobility, recent faint or fall (within the month before inclusion)

^a^Characteristics are described on available data. Missing data concerned less than 8.5% of patients

### Characteristics of ADEs

A total of 148 ADEs were collected.

In the intervention condition, 83.1% (69/83) of ADEs were reported by patients compared to 80.0% (52/65) in the control condition. Considering ADEs reported only by patients (not reported by the GP as well), more ADEs were reported in the intervention condition (68.7%; 57/83) than in the control condition (52.3%; 34/65). The three most frequent symptoms reported were dizziness, fatigue, and leg oedema (Table 5).

**Table 5:** Characteristics of adverse drug events (*N* = 148)

	Control (***N*** = 65)	Intervention (***N*** = 83)
***ADEs reported by the patient and/or his GP***^**a**^		
ADE reported by GPs only, % (n)	20.0 (13)	16.8 (14)
ADE reported by patients only, % (n)	52.3 (34)	68.7 (57)
ADE reported by GPs and patients, % (n)	27.7 (18)	14.5 (12)
ADE total, % (n)	100.0 (65)	100.0 (83)
***Symptoms***^**b**^		
Dizziness or light-headedness or Syncope (fainting) or vertigo, % (n)	30.8 (20)	12.1 (10)
Fatigue or asthenia or drowsiness, % (n)	23.1 (15)	15.7 (13)
Leg oedema, % (n)	15.4 (10)	12.1 (10)
Abdominal discomfort or diarrhoea or gastric irritation (upset stomach) or abdominal pain or nausea or vomiting or dyspepsia, % (n)	15.4 (10)	9.7 (8)
Restlessness or nervousness or anxiety or depression, % (n)	13.9 (9)	9.6 (8)
Respiratory disorder, % (n)	9.2 (6)	14.5 (12)
Myalgia (muscle pain) or muscle spasm, % (n)	7.7 (5)	7.2 (6)
Skin rashes, % (n)	3.1 (2)	9.6 (8)
Other symptoms, % (n)	44.7 (29)	38.6 (32)
Symptoms total, % (n)	100.0 (100)	100.0 (107)

^a^ ADEs reported by GPs in the study online form but not reported by patients to the research assistant (GPs only), ADEs reported by patients to the research assistant but not by the GPs in the online form (Patients only) and ADEs reported both by the GPs and by their patients

^b^ One or more symptoms could be described for one AD. *ADE* adverse drug event

### Impact of the program on ADE reporting

The proportion of patients self-reporting at least one ADE to their GP was 2.4% (13/549) in the control condition and 5.7% (31/546) in the intervention condition. The proportion of patients with at least one ADE reported either by the patient and/or by his GP was 9.3% (51/549) in the control condition and 12.3% (67/546) in the intervention condition.

#### Impact on patient self-reporting of ADEs to their GP

The results of the analysis adjusted for the period (*N* = 1095 patients) showed that the program was significantly associated with an increase in the reporting of at least one ADE by patients to their GP (odds ratio [OR] = 3.9, 95% confidence interval [CI^95^] [1.4–11.2], *p* = 0.01). After adjustment for the period, age, sex, educational level, morbidity, situations with a risk of ADEs and antihypertensive prescription (*N* = 908 patients for which data were available), the effect was still significant (adjusted odds ratio [aOR] = 3.5, CI^95^ [1.2–10.1], *p* = 0.02). The modification and the initiation of antihypertensive treatment were also significantly associated with the reporting of ADEs (aOR = 4.4, CI^95^ [1.9–10.0], *p* < 0.001 and aOR = 11.0, CI^95^ [4.6–26.4], *p* < 0.001, respectively). The proportion of patients self-reporting at least one ADE tended to decrease slightly with the period but this effect was not statistically significant (aOR = 0.8, CI^95^ [0.6–1.2], *p* < 0.31) (Table 6).

**Table 6:** Impact of the program on the reporting of at least one adverse drug event by patients, and by patients and/or General Practitioners (*N* = 908 patients)

	Patient self-reporting of ADE			Patient and/or GP reporting of ADE		
	aOR	95% CI	*p-value*	aOR	95% CI	*p-value*
Program	3.5	1.2–10.1	***0.02***	2.5	1.3–5.1	***0.01***
Period^a^	0.8	0.6–1.2	*0.31*	0.7	0.6–0.9	***0.02***
Age^b^	1.0	1.0–1.1	*0.06*	1.0	1.0–1.0	*0.21*
Sex M	0.9	0.5–1.8	*0.86*	0.8	0.5–1.2	*0.22*
Educational level					–	*–*
Secondary school or no diploma	1.0	–	*–*	1.0		
Vocational/High-school degree	0.8	0.4–1.9	*0.67*	1.1	0.7–1.9	*0.69*
University degree	1.5	0.6–3.4	*0.38*	1.1	0.6–1.9	*0.85*
Morbidity ≥3 chronic pathologies	0.8	0.2–3.6	*0.78*	1.4	0.6–3.1	*0.45*
Situations with a risk of ADEs	0.5	0.2–1.7	*0.28*	0.6	0.3–1.3	*0.18*
Antihypertensive prescription:						
Renewal	1.0	–	*–*	1.0	–	*–*
Modification	4.4	1.9–10.0	***< 0.001***	4.1	2.3–7.3	***< 0.001***
Initiation	11.0	4.6–26.4	***< 0.001***	17.7	8.9–35.4	***< 0.001***

*ADE* adverse drug event

Chronic pathologies: heart failure, heart rhythm disorder, anchor, myocardial infarction, obliterating arteriopathy of the lower limbs, renal artery stenosis, diabetes, cerebrovascular accident, COPD or chronic respiratory failure, asthma, cirrhosis or liver failure, renal failure (creatinine clearance < 60 ml/min), gout, cancer, depression, Parkinson

Situations presenting a risk of antihypertensive drug-related ADEs: hypotension, fever, nutritional depletion, diarrhoea, nausea, vomiting, abdominal pain, heat period, reduced mobility, recent malaise, recent fall

^a^from one period to another

^b^for a one year increase

#### Impact on patient and/or GP reporting of ADEs

The program was significantly associated with an increase in the proportion of patients for whom at least one ADE was reported by patients and/or by GPs (aOR = 2.5, IC^95^ [1.3–5.1], *p* = 0.01). The modification and the initiation of antihypertensive treatment were significantly associated with the reporting of ADEs (aOR = 4.1, IC^95^ [2.3–7.3], *p* < 0.001 and aOR = 17.7, IC^95^ [8.9–35.4], *p* < 0.001, respectively). Over the five periods, the proportion of patients for whom at least one ADE was reported decreased significantly (aOR = 0.7, CI^95^ [0.6–0.9], *p* < 0.02) (Table 6).

### Impact of the program on patient’s knowledge and satisfaction

Knowledge about antihypertensive prescription did not differ significantly between the intervention condition (3.5/15 points, SD 1.4) and the control condition (3.3/15 points, SD 1.3) and neither did patient knowledge about cardiovascular risk factors (1.6/9 points, SD 1.5 vs. 1.7/9 points, SD 1.4 respectively).

In the intervention conditions, the overall patient satisfaction concerning their GP was significantly higher (21.9/27 points, SD 3.1 vs. 20.1/27 points, SD 3.6 in the control condition; *p* < 0.001), patient satisfaction about communication with their GP was higher (10.8/12 points, SD 1.6 vs. 10.5/12 points, SD 1.5 in the control condition; *p* = 0.003) as well as satisfaction concerning the information given by their GP on cardiovascular risks and management of antihypertensive treatments (11.1/15 points, SD 2.0 vs 9.6/15 points, SD 1.5 in the control condition; *p* < 0.001).

## Discussion

The present study aimed at assessing a booklet for patients on antihypertensive drug management in order to encourage antihypertensive-treated patients to report ADEs to their GPs. Results showed that, by improving patient information and communication with GP, more patients self-reported ADEs to their GPs. The identification of ADEs by both patients and/or GPs was also improved. Initiation and modification of the prescription were shown to be most at risks for ADEs and should receive special attention. The intervention also improved patient satisfaction on GP communication and information given on high blood pressure management.

This study has several strengths. It is a large-scale study including 60 GPs and more than 1000 patients in two large French regions where the tested intervention was simple, hence a priori transferable to other clinical situations and sustainable. The interest of the stepped wedge design was previously discussed [18]. Finally, this design allowed us to show that the effect of the intervention was maintained whatever the period of the year (no effect of seasonality).

The study also has several limitations. First, the primary endpoint was the ADE self-reporting by patients to their GP, which represents an opportunity for the GP to improve antihypertensive drug management. However, we do not directly assess the effect of the intervention on the patient’s health status (fewer complications, better adherence, better control of hypertension,...). Yet, patient safety studies often choose a primary endpoint related to a frequency of ADEs. Second, due to a cluster design with randomization of GPs (and not patients), differences in the characteristics of the included patients were observed. In particular, there were more patients in the intervention condition with at least one medical situation presenting a risk of ADE. Although adjusted analyses showed more patient self-reports in the intervention condition, it cannot be excluded that other factors than the booklet participated in this improvement. Third, even if several patients of various socio-demographic origins and one linguist have been involved in the process of booklet development (see Table 1; 16), the booklet might be not adapted to all levels of health literacy which is known to influence patient ability to identify and report ADEs [22, 23]. The level of health literacy might have influenced patients’ capacity to report ADEs in both groups. Fourth, no data was collected on how the way the GPs delivered the booklet. This would have required conducting audio and/or video recordings of the handover of the booklet. Only the distribution of the booklet to the patient was checked. Finally, the results might be affected by an unbalanced Hawthorne effect (a change in behavior as a motivational response to the attention received through the research) between the intervention and the control groups, even if data collection was identical in both groups. Such bias, if present, would limits the generalizability of the results from research to clinical practice [24]. Future research should combine quantitative and qualitative approaches to improve the understanding of how such phenomena influences the results.

In the intervention group, the rate of patients experiencing at least one ADE was consistent with rates previously reported in primary care for antihypertensive treated patients [1, 2, 4]. The present finding suggests that the frequency of ADEs in patients treated with antihypertensive drugs may be underestimated without patient self-reports. Indeed, in the course of the research study, over half of ADEs were reported only by patients and not by their GPs. This discrepancy may reflect signs or symptoms which are not, according to GPs, related to the drug [25]. However, each reported ADE was validated by a study committee, limiting the risk of error. It is also possible that GPs do not systematically interview patients concerning ADEs or that patients did not systematically inform their GP of their symptoms.

A positive effect of the intervention was also observed on satisfaction concerning the information given by the GP on antihypertensive management and on GP communication [26]. The booklet had no effect, however, on patient knowledge about cardiovascular and antihypertensive-associated risks, which remained low. Of note, patient level of knowledge may have limited their use of the booklet and thus limited its effect on patient participation in ADE reporting [27]. Furthermore, it is well established that specific physician and patient communication behaviours are associated with improved patient health status, recall, treatment adherence, and satisfaction [28]. Although patient–physician communication is considered as a mediator of health care quality and patient safety [28], and the extensive research in this field, there seems to be no consensus on the best practice to improve it. This simple patient-centred information booklet on antihypertensive drug management may be sufficient to improve physician-patient interaction in clinical encounters.

Patients are able to identify ADEs. Hence, improving patient self-report of ADEs is a first step in improving drug management. This is all the more true when patients can self-medicate themselves. The fear of inducing nocebo effects is not, in our opinion, a sufficient argument to justify not informing patients about ADEs. Moreover, GPs should systematically look for ADEs when initiating or modifying an anti-hypertensive treatment.

## Conclusion

By improving patient information and communication with GP, a patient centred-information booklet on high blood pressure management can improve patient self-reporting of ADEs to their GPs. Initiation and modification of anti-hypertensive treatments were at higher risks for ADEs and should receive special attention. The booklet delivery also improved patient satisfaction on GP communication and high blood pressure management. Future research should assess if the use of a such patient centred-information tool can improve GP management of ADEs and patient’s health status [29, 30]. It could be measured through the rate of antihypertensive prescriptions of the GPs in accordance with the last clinical recommendations and the rate of high blood pressure-associated medical complications concerning the patients. Patients should be followed over a long period of time to explore the impact of the booklet on patient morbidity-mortality.

## Data Availability

The datasets used and/or analysed during the current study are available from the corresponding author on reasonable request.

## Abbreviations

ADE: Adverse drug event
GP: General practitioner
OR: Odds ratio
aOR: adjusted odds ratio
CI: Confidence interval
SD: Standard deviation

## References

[1] Gandhi TK, Weingart SN, Borus J, Seger AC, Peterson J, Burdick E, Seger DL, Shu K, Federico F, Leape LL, Bates DW (2003). Adverse drug events in ambulatory care. N Engl J Med.

[2] Taché SV, Sönnichsen A, Ashcroft DM (2011). Prevalence of adverse drug events in ambulatory care: a systematic review. Ann Pharmacother.

[3] Assiri GA, Shebl NA, Mahmoud MA (2018). What is the epidemiology of medication errors, error-related adverse events and risk factors for errors in adults managed in community care contexts? A systematic review of the international literature. BMJ Open.

[4] Gurwitz JH, Field TS, Harrold LR (2003). Incidence and preventability of adverse drug events among older persons in the ambulatory setting. JAMA.

[5] Gurwitz JH, Field TS, Judge J, Rochon P, Harrold LR, Cadoret C, Lee M, White K, LaPrino J, Erramuspe-Mainard J, DeFlorio M, Gavendo L, Auger J, Bates DW (2005). The incidence of adverse drug events in two large academic long-term care facilities. Am J Med.

[6] Runciman WB, Roughead EE, Semple SJ, Adams RJ (2003). Adverse drug events and medication errors in Australia. Int J Qual Health Care.

[7] Krein SL, Saint S, Trautner BW, Kuhn L, Colozzi J, Ratz D, Lescinskas E, Chopra V (2019). Patient-reported complications related to peripherally inserted central catheters: a multicentre prospective cohort study. BMJ Qual Saf.

[8] de Langen J, van Hunsel F, Passier A, de Jong-van den Berg L, van Grootheest K (2008). Adverse drug reaction reporting by patients in the Netherlands: three years of experience. Drug Saf.

[9] Little P, Griffin S, Kelly J, Dickson N, Sadler C (1998). Effect of educational leaflets and questions on knowledge of contraception in women taking the combined contraceptive pill: randomised controlled trial. BMJ.

[10] Little P, Somerville J, Williamson I, Warner G, Moore M, Wiles R, George S, Smith A, Peveler R (2001). Randomised controlled trial of self management leaflets and booklets for minor illness provided by post. BMJ.

[11] Little P, Dorward M, Warner G, Moore M, Stephens K, Senior J, Kendrick T (2004). Randomised controlled trial of effect of leaflets to empower patients in consultations in primary care. BMJ.

[12] Fick DM, Maclean JR, Rodriguez NA, Short L, Heuvel RV, Waller JL, Rogers RL (2004). A randomized study to decrease the use of potentially inappropriate medications among community-dwelling older adults in a southeastern managed care organization. Am J Manag Care.

[13] Roughead E, Pratt N, Peck R, Gilbert A (2007). Improving medication safety: influence of a patient-specific prescriber feedback program on rate of medication reviews performed by Australian general medical practitioners. Pharmacoepidemiol Drug Saf.

[14] Dawes MG, Kaczorowski J, Swanson G, Hickey J, Karwalajtys T (2010). The effect of a patient education booklet and BP “tracker” on knowledge about hypertension. A randomized controlled trial. Fam Pract.

[15] de Bont EG, Alink M, Falkenberg FC, Dinant GJ, Cals JW (2015). Patient information leaflets to reduce antibiotic use and reconsultation rates in general practice: a systematic review. BMJ Open.

[16] Keriel-Gascou M, Badet-Phan A, Le Pogam MA, Figon S, Letrilliart L, Gueyffier F, Chanelière M, Buchet-Poyau K, Duclos A, Colin C (2013). Information and active patient participation using an interactive booklet in the prescription of antihypertensive drugs in primary care. Santé Publique.

[17] Keriel-Gascou M, Buchet-Poyau K, Duclos A, Rabilloud M, Figon S, Dubois JP, Brami J, Vial T, Colin C (2013). Evaluation of an interactive program for preventing adverse drug events in primary care: study protocol of the InPAct cluster randomised stepped wedge trial. Implement Sci.

[18] Keriel-Gascou M, Buchet-Poyau K, Rabilloud M, Duclos A, Colin C (2014). A stepped wedge cluster randomized trial is preferable for assessing complex health interventions. J Clin Epidemiol.

[19] Lainer M, Vögele A, Wensing M, Sönnichsen A (2015). Improving medication safety in primary care. A review and consensus procedure by the LINNEAUS collaboration on patient safety in primary care. Eur J Gen Pract.

[20] Hussey MA, Hughes JP (2007). Design and analysis of stepped wedge cluster randomized trials. Contemp Clin Trials.

[21] Hemming K, Taljaard M, McKenzie JE (2018). Reporting of stepped wedge cluster randomised trials: extension of the CONSORT 2010 statement with explanation and elaboration. BMJ.

[22] Zhang NJ, Terry A, McHorney CA (2014). Impact of health literacy on medication adherence: a systematic review and meta-analysis. Ann Pharmacother.

[23] Mosher HJ, Lund BC, Kripalani S, Kaboli PJ (2012). Association of health literacy with medication knowledge, adherence, and adverse drug events among elderly veterans. J Health Commun.

[24] Sedgwick P, Greenwood N (2015). Understanding the Hawthorne effect. BMJ.

[25] Rosendal M, Jarbøl DE, Pedersen AF, Andersen RS (2013). Multiple perspectives on symptom interpretation in primary care research. BMC Fam Pract.

[26] Lang S, Velasco Garrido M, Heintze C (2016). Patients’ views of adverse events in primary and ambulatory care: a systematic review to assess methods and the content of what patients consider to be adverse events. BMC Fam Pract.

[27] van Beusekom MM, Grootens-Wiegers P, Bos MJ, Guchelaar HJ, van den Broek JM (2016). Low literacy and written drug information: information-seeking, leaflet evaluation and preferences, and roles for images. Int J Clin Pharm.

[28] Rao JK, Anderson LA, Inui TS, Frankel RM (2007). Communication interventions make a difference in conversations between physicians and patients: a systematic review of the evidence. Med Care.

[29] Weingart SN, Hamrick HE, Tutkus S, Carbo A, Sands DZ, Tess A, Davis RB, Bates DW, Phillips RS (2008). Medication safety messages for patients via the web portal: the MedCheck intervention. Int J Med Inform.

[30] Weingart SN, Carbo A, Tess A, Chiappetta L, Tutkus S, Morway L, Toth M, Davis RB, Phillips RS, Bates DW (2013). Using a patient internet portal to prevent adverse drug events: a randomized, controlled trial. J Patient Saf.

